# Effect of dimethyl sulfoxide on the resin-dentin bond strength of universal adhesives after thermocycling

**DOI:** 10.3389/fdmed.2026.1848702

**Published:** 2026-05-29

**Authors:** Muhammet Hilmi Kotan, Yusuf Ziya Bayındır

**Affiliations:** 1 Department of Restorative Dentistry, Faculty of Dentistry, Aksaray University, Aksaray, Türkiye; 2 Department of Restorative Dentistry, Faculty of Dentistry, Atatürk University, Erzurum, Türkiye

**Keywords:** aging, dentin, dimethyl sulfoxide, microtensile, solvent

## Abstract

**Introduction:**

The aim of this study was to evaluate the effect of DMSO pretreatment on the resin-dentin bond strength of universal adhesives after thermocycling.

**Methods:**

Seventy-two extracted human permanent molars were used and randomly assigned to 12 groups (*n* = 6). Two universal adhesives [Prime&Bond Universal (PBU) and Scotchbond Universal Plus (SBUp)], two application modes [Etch-and-Rinse (E&R) and Self-Etch (SE)], and three pretreatments (Control, 50% DMSO, and 99.99% DMSO) were investigated. Occlusal enamel was removed, and dentin surfaces were exposed and polished. Control groups were treated according to the manufacturers' instructions, while the experimental groups received additional DMSO pretreatment. Composite resin was placed incrementally using a silicone mold and light-cured. Resin-dentin beams and plates were prepared, and all specimens were subjected to thermocycling. Microtensile bond strength (µTBS) was measured using a microtensile tester. Data were analyzed using three-way ANOVA and Tukey *post-hoc* tests (*p* < 0.05).

**Results:**

Significant differences were observed among application mode (*p* < 0.001), pretreatment (*p* = 0.016), and adhesive*mode interaction (*p* = 0.029). The highest µTBS value was observed in the SBUp–E&R 99.99% DMSO group.

**Conclusion:**

Pretreatment with 99.99% DMSO significantly increased the µTBS of SBUp applied in the E&R mode compared with the control group, whereas 50% DMSO pretreatment did not produce a statistically significant increase. In contrast, DMSO pretreatments did not significantly enhance the µTBS of universal adhesives applied in the SE mode.

## Introduction

Universal adhesives are widely used by dental professionals due to their simplified application procedures, reduced technique sensitivity, and multi-mode bonding capability. Nonetheless, the bond durability of the hybrid layer produced by universal adhesive systems remains an important topic of investigation (1).

The majority of universal adhesives available on the market contain 10-methacryloyloxydecyl dihydrogen phosphate (10-MDP), a functional monomer known for its chemical affinity to hydroxyapatite, which contributes to the formation of a more stable hybrid layer (2). Water plays a crucial role in adhesive formulations by maintaining 10-MDP in its ionized state and enabling effective interaction with hydroxyapatite (3). However, although water is essential during the bonding process, residual water retained within the hybrid layer may contribute to long-term hydrolytic degradation of collagen and resin components over time (4).

For this reason, strategies aimed at improving hybrid layer stability have become an important focus in adhesive dentistry.

Effective strategies are still needed to improve the long-term durability of resin-dentin bonding (5). In this context, dimethyl sulfoxide (DMSO) has recently been introduced into adhesive dentistry as a pretreatment strategy to reduce interfacial degradation and improve hybrid layer stability. Research has demonstrated that even low concentrations of DMSO are effective in mitigating the reduction of bond strength following aging (6, 7). This may indicate that higher DMSO concentrations influence dentin bonding differently (8).

Although numerous studies have investigated DMSO pretreatment, particularly in etch-and-rinse (E&R) adhesive systems, data regarding self-etch (SE) and universal adhesive systems remain limited (9). This study aimed to assess the effects of dimethyl sulfoxide (DMSO) pretreatment on the resin-dentin bond strength of universal adhesives after thermocycling, along with an analysis of specimen failure modes and hybrid-layer micromorphology.

The following hypotheses were tested:

1. DMSO pretreatment at different concentrations does not affect the resin-dentin bond strength of universal adhesives applied in etch-and-rinse mode after thermocycling.
2. DMSO pretreatment at different concentrations does not affect the resin-dentin bond strength of universal adhesives applied in self-etch mode after thermocycling.

## Materials and methods

This research was financially supported by the Scientific Research Projects Coordination Unit of Atatürk University (Project No: TDH-2024-13769) and received ethical clearance from the Ethics Committee of the Faculty of Dentistry at Atatürk University (Decision No. 71, dated December 27, 2023; Session No. 12/2023). The experimental work was conducted at the Research Laboratories of the Faculties of Dentistry at both Atatürk University and Recep Tayyip Erdoğan University, as well as at the High Technology Research Center (YUTAM) of Erzurum Technical University. Within this investigation, two universal adhesives were applied to dentin surfaces under three distinct pretreatment conditions and two application modes, followed by assessment of their micro-tensile bond strength. The laboratory workflow included specimen preparation and the fabrication of resin-dentin beams and slabs, artificial aging of these specimens, micro-tensile bond strength testing, analysis of failure modes, and scanning electron microscopy imaging.

### Sample size calculation

Sample size calculation was performed using G*Power software version 3.1.9.7 (Heinrich Heine University, Düsseldorf, Germany). Based on a statistical power of 0.95, an effect size of 0.29, and a significance level of 0.05, sample size estimation was performed according to the planned number of teeth per experimental group.

### Tooth collection and storage

Seventy-two extracted sound human permanent molars, free from caries, restorations, and fluorosis, were collected for this study. Residual soft and hard tissues on the tooth surfaces were meticulously removed using a periodontal scaler (Scaler, H6/H7; Hu-Friedy; Chicago, IL, USA), followed by thorough cleaning with a brush under running water to ensure the removal of all debris. The specimens were then stored in distilled water at 23 ± 1 °C for a maximum duration of 2 months prior to experimentation, with the distilled water being refreshed at weekly intervals.

### Sample preparation

To ensure specimen standardization and accurate positioning, a wax block was initially crafted to fit the holder arm of the IsoMet saw. A heavy-body silicone impression was then taken from this wax block, serving as a mold for specimen preparation throughout the study. Each tooth was embedded in acrylic resin (SC, IMICYRYL, Konya, Turkey) with the enamel-cementum junction left exposed. The coronal enamel was removed using an IsoMet cutting machine (Isomet 1000, Buehler Ltd., USA) equipped with a 300-rpm diamond saw blade (#19-125 Sintered Diamond Wafering Blade; Metkon Instruments Inc., Bursa, Turkey) operating at low speed until a flat dentin surface, free of enamel, was achieved. Thereafter, the dentin surfaces were polished under running water using 320-grit silicon carbide (SiC) paper for 30 s, followed by 600-grit SiC paper for an additional 30 s to produce a consistent smear layer.

### Adhesive and restorative procedures

**Figure 1:**
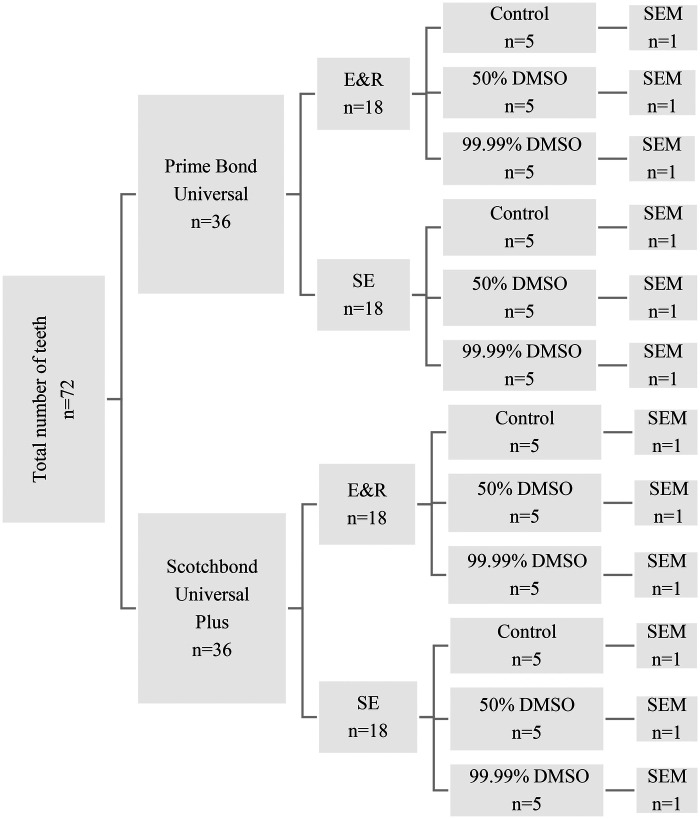
The allocation of the teeth utilized in the present study.

Seventy-two molars were randomly allocated into twelve groups (*n* = 6). The experimental design incorporated three factors: (i) two universal adhesives [Prime Bond Universal (PBU) and Scotchbond Universal Plus (SBUp)], (ii) two application modes [Etch-and-Rinse (E&R) and Self-Etch (SE)], and (iii) three pretreatment protocols [Control, 50% DMSO, and 99.99% DMSO]. The distribution of the teeth used in the present study is presented in (Figure 1).

All adhesive and restorative procedures were performed by a single operator following a pilot study conducted prior to the main experiments to standardize specimen preparation, adhesive application timing, and restorative procedures. In the control groups, the adhesive protocols were strictly adhered to according to manufacturers' guidelines, whereas the pretreatment groups received an additional application of DMSO. For the etch-and-rinse groups, Scotchbond Universal Etchant (3M ESPE, St. Paul, MN, USA), containing 34% orthophosphoric acid, was applied to the dentin surfaces for 15 s. After rinsing and gentle air drying, the DMSO pretreatment groups were treated with either 50% DMSO/water (w/w) or 99.99% DMSO. The solutions were administered using a microbrush with gentle circular motions under slight pressure for 60 s. The 50% DMSO/water solution was prepared by mixing equal volumes of 99.99% pure DMSO (Aromel Kimya, Germany) and distilled water in an Eppendorf tube, followed by vortexing for 60 s prior to use. The DMSO was applied directly from the commercial product. Subsequently, the dentin surface was gently dried with microfilter paper. Prime Bond Universal (Dentsply Sirona; Konstanz, Germany) or Scotchbond Universal Plus (3M Oral Care; Seefeld, Germany) adhesives were then applied. After light curing for 10 s using a curing unit (1,200–1,300 mW/cm^2^, D-Light Duo, GC, Tokyo, Japan), a silicone cylindrical mold with a central hole was positioned over the dentin. G-aenial Posterior A3 composite resin (GC, Tokyo, Japan) was placed in two increments of 2 mm each, achieving a total thickness of 4 mm, with each layer light-cured for 10 s. The materials used in this study, along with their compositions, pH values, and LOT numbers, are summarized in Table 1, whereas the adhesive protocols applied to each group are detailed in (Table 2).

**Table 1:** The materials used in the present study, including their brands, pH values, and LOT numbers.

Materials	Manufacturer	pH	Composition	Lot number
Prime Bond Universal (PBU)	Dentsply Sirona; Konstanz, Germany	2.7	10-MDP, bisacrylamide monomers, PENTA, isopropanol, water, initiator, stabilizer	2310000715
Scotchbond Universal Plus (SBUp)	3M, Oral Care; Seefeld, Germany	2.7	10-MDP, HEMA, 1,3-benzenediol 2-(2-hydroxyethoxy)ethyl 3-hydroxypropyl diethers, 2-methyl-2-propenoic acid, 3-triethoxysilyl propyl ester, silica and reaction products with APTES, ethanol, water, CQ, acrylic and itaconic acid copolymer, copper(II) acetate monohydrate	10517802
Scotchbond Universal Etchant	3M ESPE, St. Paul, MN, USA	0.1	34% Orthophosphoric Acid Gel	10119576
G-aenial Posterior Composite (A3)	GC, Tokyo Japan	–	Urethane dimethacrylate, dimethacrylate silicone dioxide, fluoroaluminosilicate glass, fillers, pigments, initiator	2304241
Dimethyl sulfoxide (DMSO)	Aromel Kimya, Germany	–	Formula: (CH_3_)_2_SO 99.99% DMSO (dimethyl sulfoxide)	472301

10-MDP, 10-methacryloyloxydecyl dihydrogen phosphate; PENTA, dipentaerythritol penta acrylate monophosphate; HEMA, 2-hydroxyethyl methacrylate; CQ, camphorquinone; APTES, 3-aminopropyltriethoxysilane.

**Table 2:** Adhesive procedures.

Adhesives	Mode	Pretreatment		
		Control	50% DMSO	99.99% DMSO
Prime Bond Universal (PBU)	E&R	Apply 34%–37% phosphoric acid for 15 s. Rinse for at least 15 s. Remove excess moisture with gentle air-drying; do not overdry. Proceed with the self-etch (SE) protocol.	Apply 34%–37% phosphoric acid for fifteen seconds. Rinse for a minimum of 15 s. Remove excess surface moisture with gentle air-drying; do not overdry. Apply water-based 50% DMSO to the specimen using a microbrush with light pressure and circular scrubbing motion for 60 s. Blot the applied DMSO with a microfilter paper. Proceed according to the self-etch (SE) protocol.	Apply 34%–37% phosphoric acid for 15 s. Rinse for a minimum of 15 s. Remove excess moisture from the surface with gentle air-drying; do not overdry. Apply 99.99% DMSO to the specimen using a microbrush with light pressure and circular scrubbing motion for 60 s. Blot the applied DMSO with microfilter paper. Proceed according to the self-etch (SE) protocol.
	SE	Apply the adhesive to the entire preparation using a microbrush and rub it for 20 s. Gently air-dry for at least 5 s. Light-cure for 10 s.	Apply water-based 50% DMSO to the specimen using a microbrush with light pressure and circular scrubbing motion for 60 s. Blot the applied DMSO with microfilter paper. Apply the adhesive to the entire preparation using a microbrush and rub it for 20 s. Gently air-dry for at least 5 s. Light-cure for 10 s.	Apply 99.99% DMSO to the specimen using a microbrush with light pressure and circular scrubbing motion for 60 s. Blot the applied DMSO with microfilter paper. Apply the adhesive to the entire preparation using a microbrush and rub for twenty seconds. Gently air-dry for at least 5 s. Light-cure for 10 s.
Scotchbond Universal Plus (SBUp)	E&R	Apply phosphoric acid for 15 s. Rinse thoroughly with water. Dry with oil-free, moisture-free air using a cotton pellet; do not overdry. Proceed according to the self-etch (SE) protocol.	Apply phosphoric acid for 15 s. Rinse thoroughly with water. Dry using oil-free and moisture-free air with a cotton pellet; do not overdry. Apply water-based 50% DMSO to the specimen using a microbrush with light pressure and circular scrubbing motion for 60 s. Blot the applied DMSO with microfilter paper. Proceed according to the self-etch (SE) protocol.	Apply phosphoric acid for 15 s. Rinse thoroughly with water. Dry using oil-free and moisture-free air with a cotton pellet; do not overdry. Apply 99.99% DMSO to the specimen using a microbrush with light pressure and circular scrubbing motion for 60 s. Blot the applied DMSO with microfilter paper. Proceed according to the self-etch (SE) protocol.
	SE	Apply the adhesive to the entire surface using a microbrush, rubbing for 20 s. Gently air-dry for approximately 5 s until the solvent has completely evaporated. Light-cure for 10 s.	Apply water-based 50% DMSO to the specimen using a microbrush with light pressure and circular scrubbing motion for 60 s. Blot the applied DMSO with microfilter paper. Apply the adhesive to the entire surface using a microbrush, rubbing for 20 s. Gently air-dry for approximately 5 s until the solvent has completely evaporated. Light-cure for 10 s.	Apply 99.99% DMSO to the specimen using a microbrush with light pressure and circular scrubbing motion for 60 s. Blot the applied DMSO with microfilter paper. Apply the adhesive to the entire surface using a microbrush, rubbing for 20 s. Gently air-dry for approximately 5 s until the solvent has completely evaporated. Light-cure for 10 s.

### Micro-tensile bond strength testing

Following a 24-hour storage period in distilled water, the specimens were sectioned into resin-dentin beams measuring approximately 1 mm × 1 mm (cross-sectional area of 1 mm^2^) by 8 mm (length) using an IsoMet saw, incorporating both composite and dentin portions. Specimens deemed too short for reliable testing or exhibiting irregular surfaces were excluded from further analysis. In total, 300 resin-dentin beams were prepared, with five beams derived from each tooth. All specimens underwent 10,000 thermocycles as an artificial aging protocol prior to µTBS testing, followed by microtensile bond strength testing. Prior to testing, the dimensions of each beam were precisely measured with a digital caliper to calculate the cross-sectional area. Specimens were then adhered to the microtensile testing apparatus using cyanoacrylate adhesive (Akfix 705, Istanbul, Turkey). Tensile loading was applied at a crosshead speed of 0.5 mm/min until failure, with fracture forces recorded accordingly. Bond strength values (MPa) were determined by dividing the failure load (Newtons) by the specimen's cross-sectional area in mm^2^. The tooth was considered the true experimental unit for statistical analysis. Multiple resin-dentin beams obtained from the same tooth were treated as technical replicates, and the mean µTBS value per tooth was used for statistical analysis.

### Artificial aging procedure

After preparation of all specimens (resin-dentin beams and plates), the samples were divided into groups for the aging procedure. The beams and plates of each group were placed separately into gauze bags. To prevent mix-ups, acrylic numbers ranging from 1 to 12 were assigned to each bag, and each group was designated with a corresponding number. The acrylic blocks were immersed in distilled water for one week prior to thermocycling. All specimens placed in the thermocycling device underwent temperature cycles alternating between 5 °C and 55 °C, with each cycle comprising 30 s at 5 °C, 30 s at 55 °C, and a 5-second transition time between baths, resulting in a total cycle duration of 65 s. Specimens were subjected to 10,000 thermocycles as an artificial aging protocol.

### Failure mode analysis

The fracture surfaces of all specimens tested for micro-tensile bond strength were examined using a stereomicroscope (Olympus SZ4045 TRPT, Osaka, Japan) at 20× magnification. Based on these observations, the specimens were categorized according to their failure modes.

**Figure 2:**
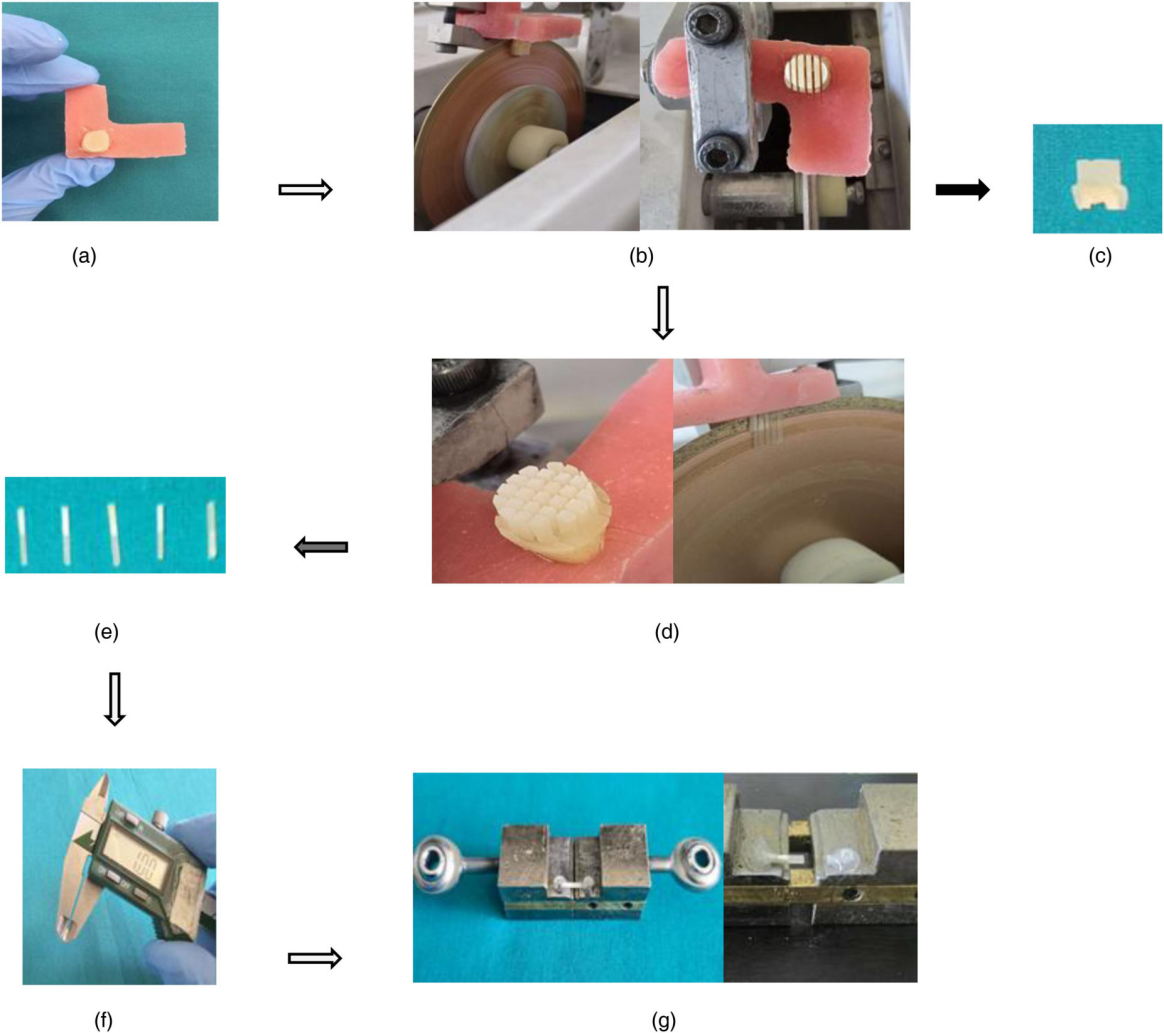
Experimental workflow of specimen preparation and analysis procedures. **(a)** Exposure of dentin tissue as a result of tooth preparation. **(b)** Resin-dentin plates were obtained following composite restoration. **(c)** Resin-dentin plate. **(d)** Preparation of resin-dentin beams. **(e)** Resin-dentin beams. **(f)** Measurement of the cross-sectional area of the resin-dentin beam. **(g)** Measurement of microtensile bond strength values.

Specimen preparation for micro-tensile bond strength testing and SEM analysis followed the workflow shown in (Figure 2**)**. Resin-dentin beams were prepared for micro-tensile bond strength testing, while resin-dentin plate specimens were prepared for SEM evaluation.

### Scanning electron microscopy (SEM) imaging

For SEM analysis, a total of 12 teeth were used, with one tooth per group. From each tooth, resin-dentin plates measuring 1 mm (thickness) × 8 mm (length) were sectioned, and only the central plate from each tooth was selected, resulting in a total of 12 resin-dentin plates. All obtained plate specimens were subjected to 10,000 thermocycles prior to SEM evaluation. After aging, the surfaces of the plates designated for imaging were polished using polishing discs from coarse to fine grit (Sof-Lex, 3M ESPE, USA), followed by a felt polishing disc (Diamond Flex, FGM Dental Group, USA). Finally, the specimens were polished with a polishing paste (Diamond Polish, Ultradent, USA). Subsequently, the specimens were ultrasonically cleaned for 3 min, then immersed in 6M hydrochloric acid for 25 s and in 6% sodium hypochlorite solution for 3 min. The samples were then dehydrated using ascending concentrations of tertiary butyl alcohol (20 min in 50%, 20 min in 95%, and 2 h in 100%). The bonded interfaces were examined using a scanning electron microscope (SEM, Quanta FEG 250, FEI).

### Statistical analysis

Statistical analyses were conducted using SPSS software version 20 (IBM, Chicago, IL, USA). Data normality and variance homogeneity were examined using the Kolmogorov–Smirnov and Levene tests, respectively. To compare overall effects, a three-way ANOVA was utilized, followed by Tukey's *post hoc* multiple comparisons. Additionally, one-way ANOVA accompanied by Tukey's *post hoc* test was applied to evaluate differences among adhesive materials and application modes collectively, as well as to investigate the impact of pretreatment within each application mode. Statistical significance was defined as *p* < 0.05 for all tests.

## Results

### Micro-tensile bond strength results

Micro-tensile bond strength values varied according to adhesive type, application mode, and pretreatment protocol. The results of the three-way analysis of variance (ANOVA) indicated significant effects of application mode (*p*<0.001), pretreatment (*p* = 0.016), and the interaction between adhesive and application mode (*p* = 0.029). No significant differences were observed in other interaction terms (*p*>0.05). The results of the Tukey multiple comparison test, on the other hand, are presented in (Table 3).

**Table 3:** Mean and standard deviation values of the groups in the present study and results of the Turkey multiple comparison test.

Adhesives	Mode	Pretreatment			*P*_1_
		Control	50% DMSO	99.99% DMSO	
Prime Bond Universal (PBU)	E&R	25.45 ± 8.10^Aa^	27.26 ± 8.17^Ba^	30.50 ± 10.24^Ba^	0.134
	SE	21.14 ± 6.27^Aa^	20.78 ± 7.26^Aa^	21.60 ± 6.14^Aa^	0.906
Scotchbond Universal Plus (SBUp)	E&R	24.62 ± 6.45^Aa^	26.33 ± 8.71^ABab^	31.28 ± 10.25^Bb^	**0.022**
	SE	24.61 ± 9.55^Aa^	24.98 ± 8.34^ABa^	25.71 ± 9.68^ABa^	0.912
*P*_2_		0.209	**0.031**	**0.001**	

Mean dentin bond strength values (MPa) and standard deviations for all groups. *P*_1_, One-way ANOVA (comparison of pretreatments); *P*_2_, One-way ANOVA (comparison of Adhesive*Application mode); E&R, etch-and-rinse; SE, self-etch. Different lowercase letters within each row and different uppercase letters within each column indicate statistically significant differences [PBU-E&R: Prime Bond Universal in Etch-and-Rinse mode; PBU-SE, Prime Bond Universal in Self-Etch mode; SBUp-E&R, Scotchbond Universal Plus in etch-and-rinse mode; SBUp-SE, Scotchbond Universal Plus in Self-Etch mode].

Bold values indicate statistically significant differences (*p* < 0.05).

Different lowercase letters within the same row indicate statistically significant differences among pretreatment groups, whereas different uppercase letters within the same column indicate statistically significant differences among adhesive/application mode groups (*p* < 0.05).

The highest micro-tensile bond strength (µTBS) value was observed in the SBUp etch-and-rinse (E&R) group treated with 99.99% DMSO (31.28 MPa), whereas the lowest value was recorded in the PBU self-etch (SE) group with 50% DMSO pretreatment (20.78 MPa). Within the SBUp-E&R subgroup, pretreatment with 99.99% DMSO resulted in significantly higher µTBS values compared with the control group (*p* < 0.05). However, no statistically significant differences were observed between the control and 50% DMSO groups or between the 50% DMSO and 99.99% DMSO groups. No significant differences were detected among pretreatment groups in the SE mode (*p* > 0.05).

### Failure mode results

To identify failure modes after the micro-tensile bond strength test, the fracture surfaces of resin-dentin beams were examined under a stereomicroscope and categorized as adhesive, composite cohesive, dentin cohesive, or mixed failures. The distribution and frequency of failure types are summarized in Table 4.

**Table 4:** Types and frequencies of failures.

Adhesives	Mode	Pretreatment	Type and frequency of failure			
			Adhesive	Mixed	Composite cohesive	Dentin cohesive
Prime Bond Universal (PBU)	E&R	Control	7	12	6	–
		50% DMSO	8	9	8	–
		99.99% DMSO	7	9	8	1
	SE	Control	12	10	3	–
		50% DMSO	12	10	3	–
		99.99% DMSO	9	12	4	–
Scotchbond Universal Plus (SBUp)	E&R	Control	8	11	6	–
		50% DMSO	6	11	7	1
		99.99% DMSO	7	8	9	1
	SE	Control	8	11	6	–
		50% DMSO	8	10	7	–
		99.99% DMSO	8	11	6	–

Adhesive failures were more frequently observed in PBU, whereas composite cohesive and dentin cohesive failures were more commonly seen in SBUp. Mixed failures were observed equally in both adhesives. The control and 50% DMSO groups of PBU-SE exhibited the highest frequency of adhesive failures. The control group of PBU-E&R and the DMSO group of PBU-SE showed the highest incidence of mixed failures, while the DMSO group of SBUp-E&R demonstrated the highest number of composite cohesive failures. Dentin cohesive failure was observed only in the DMSO group of PBU-E&R and in the 50% and DMSO groups of SBUp-E&R (Table 4). In all specimens, the distribution of failure types from most to least frequent was as follows: 41% mixed, 34% adhesive, 24% composite cohesive, and 1% dentin cohesive. When compared with bond strength values, the failure types showed good correspondence. In the E&R mode, particularly within the DMSO pretreatment groups, higher bond strength values together with the increased occurrence of dentin cohesive fractures were consistent with the µTBS findings.

### SEM analysis results

**Figure 3:**
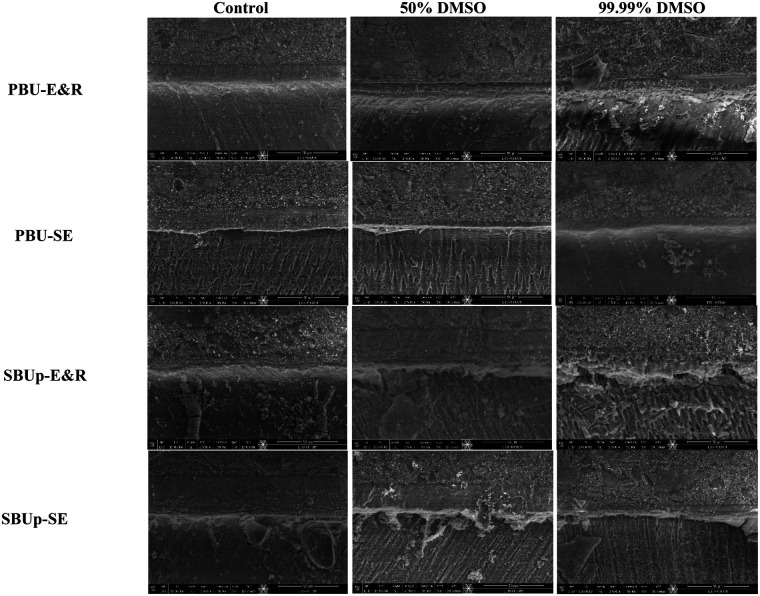
SEM images of the resin-dentin interfaces obtained at ×2,500 magnification. PBU, Prime Bond Universal; SBUp, Scotchbond Universal Plus; E&R, etch-and-rinse; SE, self-etch; DMSO, dimethyl sulfoxide.

Cross-sectional SEM images of the resin-dentin interfaces are shown in Figure 3. Distinct micromorphological features—such as hybrid layers of varying thicknesses and resin tags of different lengths—were observed. The etch-and-rinse (E&R) modes of the universal adhesives produced thicker hybrid layers compared with the self-etch (SE) modes. No differences in hybrid layer micromorphology were detected between the two universal adhesives in either the E&R or SE modes. In the PBU-E&R group, 50% DMSO pretreatment resulted in longer and more distinct resin tag formation compared with the other groups, whereas 99.99% DMSO pretreatment resulted in a higher density of resin tags per interfacial area compared with the control and 50% DMSO groups. Similarly, in the SBUp-E&R group, DMSO pretreatment produced a higher density of shorter resin tags compared with the 50% DMSO and control groups (Figure 3).

## Discussion

The findings showed that the effect of DMSO pretreatment depended on both adhesive type and application strategy. When universal adhesives were applied in the etch-and-rinse (E&R) mode with a 50% DMSO pretreatment, no statistically significant enhancement in bond strength was observed. However, the use of 99.99% DMSO significantly increased the bond strength of SBUp-E&R (*p* < 0.05), leading to the rejection of the first hypothesis. In contrast, DMSO pretreatments in the self-etch (SE) mode did not yield statistically significant improvements in bond strength (*p* > 0.05). Therefore, the second hypothesis was accepted.

Although several studies have reported beneficial effects of DMSO pretreatment on dentin bonding, the available findings remain inconsistent and appear to depend on adhesive composition, bonding strategy, and aging conditions. Mello et al. evaluated Scotchbond Universal adhesive in both E&R and SE modes following DMSO, 50% DMSO/water, and 50% DMSO/ethanol pretreatments and found no statistically significant increase in bond strength either immediately or after 6 months of aging (10). Likewise, Stape et al. reported that although DMSO-based pretreatments reduced nanoleakage and improved interfacial integrity, they did not significantly increase the bond strength of the Scotchbond Multi-Purpose adhesive system applied in the E&R mode (11). Chaharom et al. also observed no significant improvement in bond strength after DMSO pretreatment of All-Bond Universal adhesive in either E&R or SE modes, despite slight numerical increases after thermocycling (12). These findings suggest that the influence of DMSO on dentin bonding is material-dependent and closely related to adhesive composition and application strategy. Positive outcomes following DMSO pretreatment have also been reported. Cardenas et al. demonstrated increased bond strength values for universal adhesives applied to both sound and eroded dentin in E&R and SE modes after 50% DMSO/water pretreatment (13). Karadas et al. similarly observed improved bonding performance for several universal adhesives under both moist and dry dentin conditions, accompanied by reduced nanoleakage in some adhesive systems (14). Wendlinger et al. further showed that the beneficial effects of DMSO pretreatment could be maintained even after 4 years of aging (15). In addition, Zaniboni et al. and Karishma et al. reported enhanced durability and stability of resin-dentin bonds in universal adhesive systems following DMSO application (16, 17). Salim et al. also found that DMSO-containing pretreatments improved the bond strength of TheraCal to apical root dentin (18).

The conflicting findings reported in the literature may be attributed to differences in adhesive composition, solvent systems, application strategies, dentin substrate conditions, aging protocols, and bond strength testing methodologies. Variations in the concentration and solvent carrier of DMSO may also influence adhesive infiltration and hybrid layer formation differently among adhesive systems.

In the self-etch (SE) mode, universal adhesives containing 10-MDP establish a chemical bond with calcium ions, resulting in the formation of stable calcium salts (19, 20). However, phosphoric acid etching in the E&R strategy removes the smear layer and partially dissolves hydroxyapatite, potentially reducing the contribution of chemical bonding (21). Several studies have also demonstrated that universal adhesives may still achieve favorable bond strength values following phosphoric acid etching (22–25).

Hidari et al. stated that when a universal adhesive is used in the E&R mode, the 10-MDP contained within plays an important role in improving dentin bond quality and may infiltrate into the deeper regions of dentin, establishing hydrophobic interactions with the exposed collagen fibrils (26). In their study employing the STD NMR technique to explore protein–ligand interactions between various monomers and atelocollagen (a collagen model), Hiraishi et al. linked the prominent STD signals to protons located on the tail region of 10-MDP. These signals persisted even after the addition of HEMA to the 10-MDP–DMSO mixture. However, when 10-MDP was combined directly with HEMA, a significant reduction in the intensity of STD signals from 10-MDP protons was observed. The authors attributed this effect to HEMA molecules surrounding 10-MDP, thereby impeding its hydrophobic interaction with collagen (27).

In present study, the treatment of the dentin surface—where a high amount of collagen fibers are exposed as a result of acid etching—with DMSO may have dissolved the 10-MDP monomer present in the SBUp adhesive, thereby eliminating the limiting effect of HEMA. This could explain why the DMSO pretreatment group of SBUp-E&R demonstrated significantly higher bond strength values compared to the control group. In contrast, the 50% DMSO pretreatment, which contains the same volume of water, may have increased the surface water content, potentially preventing a significant increase in bond strength values.

Balakrishnan et al. also reported that DMSO pretreatment significantly improved the bond strength of Single Bond Universal adhesive in the E&R mode (28). Similar to SBUp, Single Bond Universal contains HEMA, supporting the possibility that DMSO pretreatment may enhance the interaction between HEMA-containing adhesives and exposed collagen fibrils on acid-etched dentin surfaces.

Although moist dentin is necessary for hybrid layer formation (29), excessive residual water may contribute to hydrolytic degradation over time (30, 31). DMSO may partially replace residual water within the collagen matrix and facilitate adhesive infiltration into demineralized dentin. Previous studies have demonstrated that DMSO can disrupt hydrogen bonding within collagen fibrils, alter collagen organization, improve dentin wettability, and enhance resin monomer penetration into the exposed collagen network (6–8, 32).

Although statistically significant differences were observed only in the SBUp-E&R group treated with 99.99% DMSO, DMSO pretreatments generally tended to improve bond strength values in the E&R mode. Fracture analysis also demonstrated a shift from adhesive failures toward cohesive and mixed failures in the DMSO-treated E&R groups, suggesting improved interfacial integrity. Recent randomized clinical trials have also demonstrated promising outcomes of DMSO pretreatment in cervical restorations, supporting the translational potential of DMSO-based adhesive strategies in restorative dentistry (33, 34).

The lower bond strength values observed in the PBU-SE groups may also be associated with adhesive composition and application characteristics. HEMA-free adhesives are considered more technique-sensitive during solvent evaporation and air-drying procedures. Previous studies have demonstrated that inadequate air-drying may negatively affect the bond strength of HEMA-free self-etch adhesives (35, 36). Since the manufacturer recommends only gentle air-drying for PBU, incomplete solvent evaporation may have contributed to the lower bond strength values observed in the SE groups.

Additionally, the ultra-mild acidity of the universal adhesives used in this study may have limited smear layer dissolution and collagen exposure in the SE mode. Since DMSO was directly applied onto smear-covered dentin surfaces, its penetration into the collagen matrix may have been restricted, which may have limited its ability to enhance micromechanical retention (9).

SEM analysis revealed thicker hybrid layers in the E&R groups compared with the SE groups, which corresponded with the generally higher bond strength values observed in the E&R mode. DMSO pretreatment also resulted in a higher density of resin tags per interfacial area within the adhesive interface, although these tags appeared shorter in some groups. Previous studies have suggested that hybrid layer quality may correlate more strongly with bond strength than resin tag length alone (37, 38).

The present study has several limitations. Intrinsic variations among dentin substrates and the preparation of multiple microtensile specimens from extracted teeth may have influenced bond strength outcomes despite standardization procedures. In addition, the limited disclosure of adhesive compositions by manufacturers restricts interpretation of the exact chemical interactions responsible for the observed results. Future studies incorporating longer aging periods, incubator storage conditions, Raman spectroscopy, and nanoleakage analysis may further clarify the mechanisms and long-term effects of DMSO pretreatment on universal adhesive systems.

## Conclusions

Within the limitations of this study, 50% DMSO pretreatment did not significantly improve the bond strength of either adhesive applied in the etch-and-rinse mode. In contrast, 99.99% DMSO significantly increased the bond strength of Scotchbond Universal Plus applied in the etch-and-rinse mode, whereas no significant improvement was observed for Prime&Bond Universal. Regardless of concentration, DMSO pretreatments did not significantly affect the bond strength of adhesives applied in the self-etch mode.

## Data Availability

The original contributions presented in the study are included in the article/Supplementary Material, further inquiries can be directed to the corresponding author.
